# Effects of the Oat Hay Feeding Method and Compound Probiotic Supplementation on the Growth, Antioxidant Capacity, Immunity, and Rumen Bacteria Community of Dairy Calves

**DOI:** 10.3390/antiox12101851

**Published:** 2023-10-12

**Authors:** Yong-Qing Guo, Ya-Ru Hu, Su-Ran Liu, Meng Wang, Zhen-Yu Xian, De-Wu Liu, Bao-Li Sun, Yao-Kun Li, Guang-Bin Liu, Ming Deng, Wen-Feng Hu, Qing-Shen Liu

**Affiliations:** 1 College of Animal Science, South China Agricultural University, Guangzhou 510642, China; yongqing@scau.edu.cn (Y.-Q.G.); huyaru9712@163.com (Y.-R.H.); abc1452118779@126.com (S.-R.L.); 17697215935@163.com (M.W.); z840432229@163.com (Z.-Y.X.); dwliu@scau.edu.cn (D.-W.L.); baolisun@scau.edu.cn (B.-L.S.); ykli@scau.edu.cn (Y.-K.L.); gbliu@scau.edu.cn (G.-B.L.); dengming@scau.edu.cn (M.D.); 2 College of Food Science, South China Agricultural University, Guangzhou 510642, China; wfhu@scau.edu.cn

**Keywords:** calf, oat hay, probiotics, antioxidant capacity, immunity, rumen bacteria community

## Abstract

This study aimed to investigate the effects of the oat hay feeding method and compound probiotics (CMP) on the growth, health, serum antioxidant and immune indicators, rumen fermentation, and bacteria community of dairy calves from 3 to 5 months of age. Forty-eight female Holstein calves (80 ± 7 days of age, 93.71 ± 5.33 kg BW) were selected and randomly divided into four groups. A 2 × 2 factorial design was adopted for the experiment, with the factors of the oat hay feeding method (fed as free-choice or 16.7% in the diet) and compound probiotics (CMP) inclusion (0.15% or 0%) in the pelleted starter. The results showed that, compared with giving oat hay as free-choice, feeding a diet of 16.7% oat hay increased the pelleted starter intake at 1–84 d (*p* < 0.05), with an average daily gain (ADG) at 61–84 d (*p* = 0.02); adding CMP to the pelleted starter did not significantly affect body weight, and reduced the fecal index (*p* < 0.05). Feeding 16.7% oat hay increased the concentration of IgA, IgG, and IgM (*p* < 0.01), while adding CMP increased the catalase (*p* < 0.01) and decreased the concentration of malondialdehyde (*p* < 0.01) in serum. Feeding 16.7% oat hay increased the ruminal concentration of propionic acid (*p* < 0.05) and isobutyric acid (*p* = 0.08), and decreased the ruminal pH (*p* = 0.08), the concentration of acetic acid (*p* < 0.05), and the ratio of acetic acid to propionic acid (*p* < 0.01). Feeding 16.7% oat hay reduced the relative abundance of ruminal *Firmicutes*, *Unidentified-Bacteria, Actinobacteria*, *Prevotella*, *NK4A214-group*, *Olsenella*, and *Actinobacteriota* (*p* < 0.05); adding CMP increased the relative abundance of ruminal *Prevotella*, *Rikenellaceae-RC9-gut-group*, *Ruminococcus*, *NK4A214-group*, and *Ruminococcus* (*p* < 0.05), and decreased the abundance of *Desulfobacterora*, *Prevotella-7*, and *Erysipelotricaceae-UCG-002* (*p* < 0.05). In conclusion, feeding a diet of 16.7% oat hay increased the pelleted starter intake and average daily gain, while slightly reducing the ruminal pH values; adding CMP to the pelleted starter resulted in reduced diarrhea incidence, increased serum antioxidant capacity and immunity, as well as ruminal richness and diversity of microorganisms in dairy calves from 3 to 5 months of age.

## 1. Introduction

The health and growth performance of calves are not only related to the economic benefits of producers, but also related to the stable growth of dairy herds and the sustainable development of dairy farms [1]. Research has shown that early nutritional regulation and feeding management can improve the growth, development, and health of calves [2]. Feeding high-level starter is helpful to rumen development, as the carbohydrates in it can produce butyric acid and propionic acid [3]. However, excessive readily fermentable carbohydrates can cause a rapid decrease in ruminal pH, leading to ruminal acidosis [4]. Studies have shown that providing hay may help to avoid ruminal and hindgut acidosis in calves [5]. Adequate structural fiber in calves’ diet is crucial for stimulating rumen papilla development, chewing activity, and saliva secretion, which are necessary for healthy rumen and gut function [6]. Therefore, it is suggested that a certain amount of hay should be supplemented in calves’ diet. Some studies have investigated the effects of hay restriction on calves, but the results are inconsistent. Nemati et al. [7] provided 25% alfalfa hay for calves of 7–10 weeks, and their growth rate reached 900 g/d. Meta-analysis showed that calves fed with high-level forage gained more weight on average than calves fed with low-level forage [8]. Further research is needed to determine whether restricting hay feeding can affect the growth and development, gastrointestinal health, antioxidant and immune status of weaned calves.

There are complex microbial communities in the digestive tract of animals, and the health of the animals is highly correlated with the microbial communities in the gut [9]. Previously, antibiotics were widely used to prevent and treat gastrointestinal infections in livestock. However, antibiotic resistance had a long-term impact and caused damage to intestinal flora [10,11]. In 2014, the International Scientific Association for Probiotics and Prebiotics (ISAPP) emphasized the importance of probiotics in improving animal viability [12]. Microecological preparation includes probiotics, prebiotics, and synbiotics, which have beneficial effects on the host when applied in sufficient amounts [13]. After weaning, the digestive physiological function of the developing calves changes sharply. Many factors such as diet composition, growth, and development at this stage directly affect future productivity [14]. Probiotic supplementation in this period is helpful to reduce the exposure of developing rumen to harsh conditions, and to reduce the adverse effects on health and growth caused by weaning stress [15]. Since the beneficial effects of probiotics are strain dependent, it has been suggested that combinations of different probiotic strains may be more effective than single-strain probiotics [16].

According to the above literature, there is still controversy regarding the optimal hay level and feeding method in the diet of weaned dairy calves. In addition, further research is needed to determine whether there is a corresponding interaction between hay feeding methods and the addition of probiotics to the pelleted starter. Therefore, in this study, we hypothesized that restricted feeding of oat hay and compound probiotic supplementation can improve the growth performance and health of weaned calves. Based on these hypotheses, this study aimed to examine the effect and the mechanism of oat hay restriction and compound probiotics on growth, health, serum antioxidant and immune indicators, rumen fermentation, and bacteria communities of dairy calves from 3 to 5 months of age.

## 2. Materials and Methods

### 2.1. Animals and Experimental Design

The experiment was conducted from November to February at the experimental dairy farm of the South China Agricultural University (Hezhou, China). In this experiment, 48 weaned female Holstein calves (80 ± 7 days of age, 93.71 ± 5.33 kg of initial body weight; mean ± SE) were selected, and randomly divided into four treatment groups (*n* = 12). The study lasted for 84 days. Before the experimental period, all calves were fed the chopped oat hay and pelleted starter as free-choice. In the trial period, a 2 × 2 factorial design was adopted for the experiment, with the factors of the oat hay feeding method and compound probiotic (CMP) product inclusion in the pelleted starter. Dietary treatments were as follows: (1) chopped oat hay and pelleted starter fed as free-choice (F); (2) chopped oat hay and pelleted starter fed as free-choice, with 0.15% CMP added to the pelleted starter on an air dry basis (FP); (3) diet with 16.7% chopped oat hay and 83.3% pelleted starter on an air dry basis (L); (4) diet with 16.7% chopped oat hay and 83.3% pelleted starter, with 0.15% CMP added to the pelleted starter (LP). In order to facilitate feeding, weaned calves on scaled dairy farms in South China are usually fed with a 1:4 ratio of hay to pelleted starter. After calculation, the proportion of hay was 16.7%, and that of pelleted starter was 83.3%. The CMP product used in this experiment contained inactivated *Lactobacillus acidophilus* 10^7^ cell count/g, *Bacillus subtilis* 10^7^ colony-forming unit [CFU]/g, and *Aspergillus oryzae* 10^7^ u/g, provided by Bioforte Biotechnology (Shenzhen) Co., Ltd. (Shenzhen, China). The ingredients and nutritional composition of the pelleted starter are listed in Table 1. The oat hay was cut into 3–4 cm lengths for feeding, and the nutritional components are listed in Table 1. The calves were fed with pelleted starter and oat hay twice daily at 8:00 am and 15:00 pm. The pelleted starter and oat hay were fed separately in two troughs in each pen. The pens were bedded with bamboo chaff and were refreshed every day. Manure was removed daily to keep the pens clean and dry. No animals were sick or were treated for sicknesses or vaccinated during the trial. The calves had free access to water. During the study, the temperature of the pens was 10–17 °C and the air humidity was 70–75%.

### 2.2. Feed Intake and Growth Performance

During the trial period, the feed amount of pelleted starter and oat hay for each group of experimental calves was recorded daily, and the residual feed was collected and weighed before each morning feeding to calculate the intake of pelleted starter and oat hay. All the calves were weighed and recorded on day 0, day 30, day 60, and day 84 of the trial period before morning feeding.

Pelleted starter and oat hay were collected every 10 days, dried in an oven (Model 2000; Experimental Mill, Beijing, China) at 65 °C for 48 h, then ground through a 1 mm screen using a Wiley mill (standard model 4; Arthur H. Thomas Co., Philadelphia, PA, USA). The dry matter (DM), crude protein (CP), ether extract (EE), calcium, and phosphorus were determined according to the Association of Official Analytical Chemists method [17]. The neutral detergent fiber (NDF) and acid detergent fiber (ADF) content were determined using the ANKOM A-200i fiber analyzer (Ankom Technology Corp., Fairport, NY, USA) according to the method of Van Soest et al. [18]. Starch was determined by colorimetry on a Synergy H1 Microplate Reader (BioTeck, San Leandro, CA, USA) [19].

### 2.3. Diarrhea Incidence

The calves’ feces were scored before morning feeding each day from day 31 to day 60 of the experiment. Briefly, a standard scoring procedure (1 = normal feces; 2 = semi-formed feces; 3 = loose feces; and 4 = watery feces) were used by two researchers who were blinded to the experimental groups. Diarrhea was recorded when the calf feces score was ≥3. The onset and duration of diarrhea were recorded. The diarrhea rate was calculated according to the procedure described by Sun et al. [20], and the formula was given as: diarrhea rate = (number of calves with diarrhea × days of diarrhea)/(total number of calves × examined days) × 100. The fecal consistency index (FCI) proposed by Marcondes et al. [21] was employed. The FCI was calculated at different stages of the experiment to judge the softness versus hardness of feces, as follows:

$$\mathrm{FCI}=\frac{\left(dE1\times 1)+(dE2\times 2)+(dE3\times 3)+(dE4\times 4\right)}{Td\times 4}.$$

In the formula, dE1, dE2, dE3, and dE4 represent the days when the feces score was 1, 2, 3, and 4, respectively. Td represents the test evaluation days.

### 2.4. Blood Sampling and Analysis

Before the morning feeding on the 84th day of the trial period, 10 mL of blood was collected from the jugular vein of all calves, and the blood samples were left standing for 30 min and centrifuged at 3000 r/min for 15 min. The serum was collected into a 1.5 mL centrifuge tube, and stored at −20 °C for further analyses. The level of glutathione peroxidase (GSH-Px), superoxide dismutase (SOD), total antioxidant capacity (T-AOC), catalase (CAT), and malondialdehyde (MDA) in the serum were detected with kits from Nanjing Jiancheng Bioengineering Institute (Nanjing, China). The level of immunoglobulin A (IgA), immunoglobulin G (IgG), and immunoglobulin M (IgM) in the serum were determined by the enzyme-linked immunosorbent assay (ELISA) method using an enzyme-labeled analyzer (Rayto, Shenzhen, China).

### 2.5. Rumen Sampling and Analysis

Before the morning feeding on the 84th day of the trial period, 60 mL rumen fluid samples were collected from each calf with a rumen fluid oral collector (Kelibo A1164K, Wuhan, China). The samples were filtered through four layers of gauze, and the pH was immediately measured with a pH meter (Sartorius, PB-10, Gottingen, Germany). The concentration of ammoniacal nitrogen (NH_3_-N) was determined using the colorimetric method [22]. The content of volatile fatty acids (VFA) was quantified using a high-performance gas chromatograph (Aglient 7890B, Santa Clara, CA, USA) with an HP-INNOWax capillary column (30.0 m × 320 μm × 0.5 μm) and FID detector.

Extraction of the total DNA from rumen samples was performed using the CTAB method, according to the instructions provided with the commercial DNA extraction kit (Tiangen Biochemical Technology Company, Beijing, China). Selection of the V4 region of the 16SrDNA gene was conducted by PCR amplification. The universal primers were 515F (5’-GTGCCAGCMGCCGCGGTAA-3’-3’) and 806R (5’-GGACTACHVGG GTWTCTAAT-3’). All PCR reactions were carried out in 30 µL reactions with 15 µL of Phusion^®^ High-Fidelity PCR Master Mix (New England Biolabs, Beijing, China), 0.2 µM of forward and reverse primers, and about 10 ng of template DNA. The PCR products were sequenced by equimolar paired-end sequencing on the Illumina Novaseq 6000 platform (Personal Biotechnology Co., Ltd., Shanghai, China). Sequences analysis was performed with Uparse software (Uparse v7.0.1001) [23]. Sequences with ≥97% similarity were assigned to the same OTUs. A representative sequence for each OTU was screened for further annotation. For each representative sequence, the Silva Database [24] was used to annotate taxonomic information based on Mothur algorithm. In order to study the phylogenetic relationships of different OTUs, and the difference of the dominant species in different samples (groups), multiple sequence alignments were conducted using the MUSCLE software (Version 3.8.31) [25]. OTU abundance information was normalized using a standard of sequence numbers corresponding to the sample with the fewest sequences.

The α- and β-diversity of the bacterial communities were calculated with the QIIME (Version 1.7.0) software and the “vegan” package in R (Version 2.15.3) software [26], and β-diversity was analyzed with the “vegan” package in R software(version 4.2.1). The function of the bacterial community was predicted from the PICRUST database [27]. Linear discriminant analysis (LDA) effect size (LEfSe) and functional prediction of the microflora were carried out using an online tool (http://huttenhower.sph.harvard.edu/galaxy/) accessed on 15 September 2022, and LDA scores >2 and *p* < 0.05 were selected as cutoffs. All sequencing and analysis were performed by Novogene, Beijing, China.

### 2.6. Statistical Analysis

All results were analyzed using the MIXED procedure of SAS (version 9.4, SAS Institute Inc., Cary, NC, USA), and Tukey was used for multiple comparisons. The following model was used for statistical analysis:

Y_ij_ = μ + O_i_ + P_j_ + OP_ij_ + ε_ij_

where Y_ij_ = dependent variable, μ = population mean, O_i_ = feeding effect of oat hay, P_j_ = effect of CMP, OP_ij_ = interaction effect of oat hay and CMP, and ε_ij_ = random residual. The outliers were processed based on the absolute studentized residual values >3. The threshold of significance was set at *p* < 0.05; trends were declared at 0.05 < *p* < 0.10.

## 3. Results

### 3.1. Intake and Growth Performance

In the current study, compared with the calves fed oat hay as free-choice, feeding 16.7% oat hay increased the pelleted starter and starch intake of calves at 31–60 d, 61–84 d, and 1–84 d (*p* < 0.05), the protein intake at 31–60 d (*p* < 0.05), and the average daily gain (ADG) at 61–84 d (*p* = 0.02); however, the oat hay intake decreased at 31–60 d, 61–84 d, and 1–84 d, the NDF intake at 61–84 d and 1–84 d (*p* < 0.05) (Table 2). Feeding CMP increased the oat hay intake at 1–30 d, and the protein and starch intake at 61–84 d (*p* = 0.05); however, the pelleted starter intake decreased at 61–84 d, the total feed intake at 61–84 d, and the NDF intake at 1–30 d and 61–84 d (*p* < 0.05), but did not significantly affect the body weight and daily gain of calves. In addition, the interaction between the oat hay feeding method and the CMP on the daily gain of calves at 31–60 d and 61–84 d, the pelleted starter intake at 1–30 d and 31–60 d, the oat hay intake at 1–30 d, the total feed intake at 1–30 d, the protein and starch intake at 1–30 d and 31–60 d, and the NDF intake at 1–30 d were observed (*p* < 0.05).

### 3.2. Diarrhea Incidence

According to Table 3, the oat hay feeding method had no effect on the diarrhea rate and fecal index of calves, and the addition of CMP to the pelleted starter significantly reduced the fecal index of calves (*p* < 0.05). There was no interaction between the oat hay feeding method and CMP.

### 3.3. Serum Antioxidant and Immune Indicators

According to Table 4, compared with the calves fed oat hay as free-choice, feeding 16.7% oat hay tended to decrease the serum concentration of GSH-Px (*p* = 0.08), and increased the serum content of IgA, IgG, and IgM significantly (*p* < 0.01). The addition of CMP to pelleted starter significantly increased the serum content of CAT (*p* < 0.01) and decreased the content of MDA (*p* < 0.01). The oat hay feeding method and CMP had interactive effects on the concentration of SOD (*p* = 0.05), CAT (*p* = 0.06), and MDA (*p* = 0.01) in serum.

### 3.4. Rumen Fermentation Parameters

According to Table 5, compared with the calves fed oat hay as free-choice, feeding 16.7% oat hay increased the concentration of propionic acid (*p* < 0.05) and isovaleric acid (*p* = 0.08) in the rumen, and decreased the ruminal pH (*p* = 0.08), the concentration of acetic acid (*p* < 0.05), and the ratio of acetic acid to propionic acid (*p* < 0.01). Adding CMP to the pelleted starter did not significantly affect the rumen fermentation parameters of the calves. There was no interaction between the oat hay feeding method and CMP on the rumen fermentation parameters.

### 3.5. Rumen Bacteria Community

**Figure 1:**
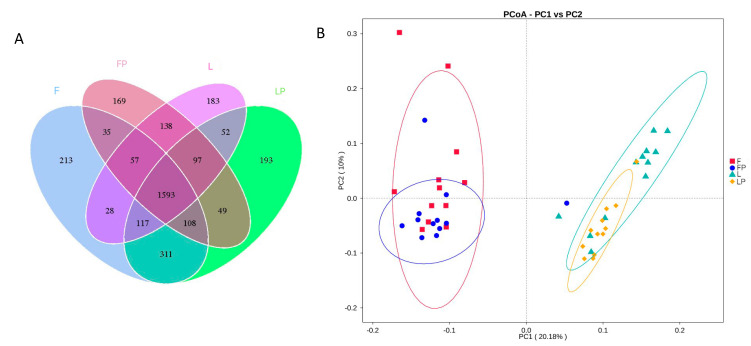
Effects of different dietary treatments on the rumen bacteria flora in dairy calves: (**A**) Venn diagram of the OTUs; (**B**) Principal Coordinates Analysis (PCoA). (F, oat hay fed as free-choice; FP, oat hay fed as free-choice, with 0.15% CMP added to pelleted starter; L, fed with a diet of 16.7% oat hay; LP, fed with a diet of 16.7% oat hay, with 0.15% CMP added to pelleted starter).

According to Figure 1A, there are 1593 OTUs shared by the four groups, which is 47.65% of the total. The number and proportion of OTUs in each experimental group were 2462 and 73.65% in the F group, 2246 and 67.19% in the FP group, 2265 and 67.75% in the L group, and 2520 and 75.38% in the LP group. There were 213, 169, 183, and 193 unique OTUs in the F, FP, L, and LP groups, respectively. According to Principal Coordinates Analysis (PCoA, Figure 1B), the first and second principal coordinates explained 20.18% and 10.00% of the variations, respectively. The samples from the calves fed oat hay as free-choice and 16.7% oat hay are far from each other, indicating that different oat hay feeding methods have a large impact on the rumen bacterial flora composition. The L group and LP group are partly overlapped, but mostly separated.

According to Table 6, compared with the calves fed oat hay as free-choice, feeding 16.7% oat hay significantly decreased the ACE, Chao1, and Shannon indices (*p* < 0.01). Adding probiotics to the pelleted starter significantly increased the ACE, Chao, and Shannon indices (*p* < 0.05). There was an interaction between the oat hay feeding method and CMP on the Simpson and Shannon indices (*p* < 0.05).

**Figure 2:**
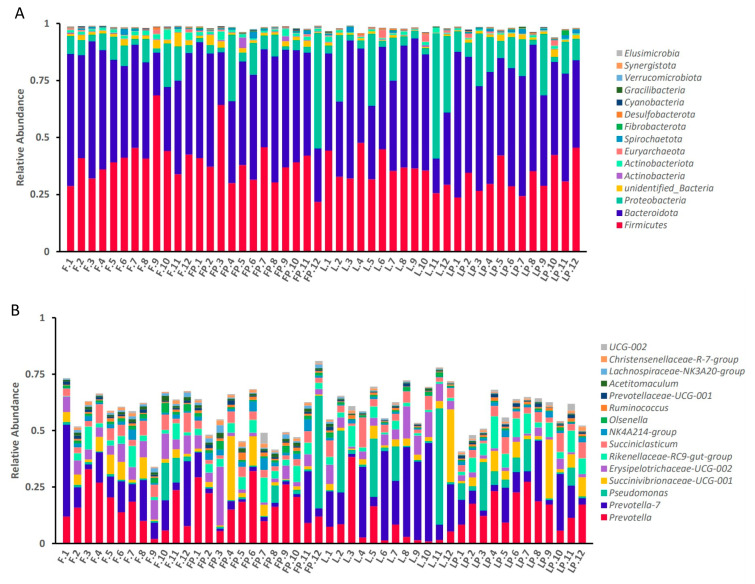
Effect of dietary treatments on an accumulation map of rumen bacteria flora in dairy calves: (**A**) at the phylum level; (**B**) at the genus level. (F, oat hay fed as free-choice; FP, oat hay fed as free-choice, with 0.15% CMP added to pelleted starter; L, fed with a diet of 16.7% oat hay; LP, fed with a diet of 16.7% oat hay, with 0.15% CMP added to pelleted starter).

At the phylum level, as shown in Table 7 and Figure 2A, feeding 16.7% oat hay reduced the relative abundance of *Firmicutes*, *Actinobacteria*, *Unidentified-Bacteria*, and *Actinobacteriota* (*p* < 0.05). Adding CMP to pelleted starter tended to increase the relative abundance of *Fibrobacterota* (*p* = 0.08) and decrease the relative abundance of *Desulfobacterora* (*p* < 0.05). The oat hay feeding method and CMP had interaction on the relative abundance of *Unidentified-Bacteria* (*p* = 0.06).

At the genus level, as shown in Table 8 and Figure 2B, feeding 16.7% oat hay increased the relative abundance of *Prevotella_7* (*p* < 0.05), and decreased the relative abundance of *Prevotella*, *NK4A214_group* and *Olsenella* (*p* < 0.05). Adding CMP to pelleted starter increased the relative abundance of *Prevotella*, *Rikenellaceae-RC9-gut-group*, Ruminococus, *NK4A214-group* and *Ruminococus* (*p* < 0.05), and decreased the relative abundance of *Prevotella-7* and *Erysipelothiaceae-UCG-002* (*p* < 0.05). There was no interaction between oat hay and CMP at the genus level.

**Figure 3:**
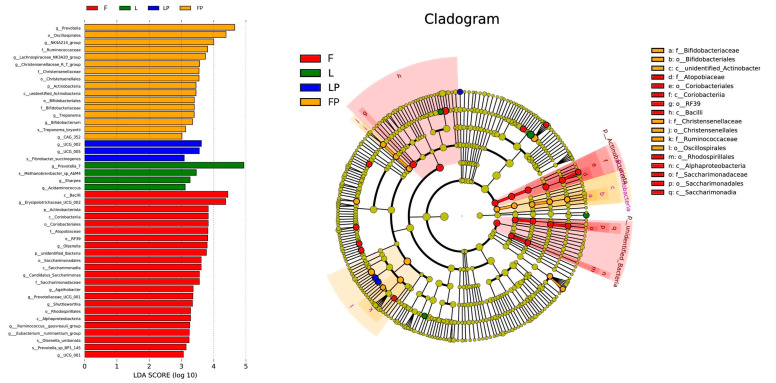
LEfSe analysis of rumen bacterial flora in dairy calves. (F, oat hay fed as free-choice; FP, oat hay fed as free-choice, with 0.15% CMP added to pelleted starter; L, fed with a diet of 16.7% oat hay; LP, fed with a diet of 16.7% oat hay, with 0.15% CMP added to pelleted starter).

LEfSe analysis showed that the 46 different bacterial strains in the four groups are (Figure 3). Twenty-three species are in the F group, 16 species in the FP group, four species in the L group, and three species in the LP group. At the class level, the *Bacilli* in the F group are highly enriched. At the order level, the FP group is highly enriched with the order of *Oscillospirales*.

**Figure 4:**
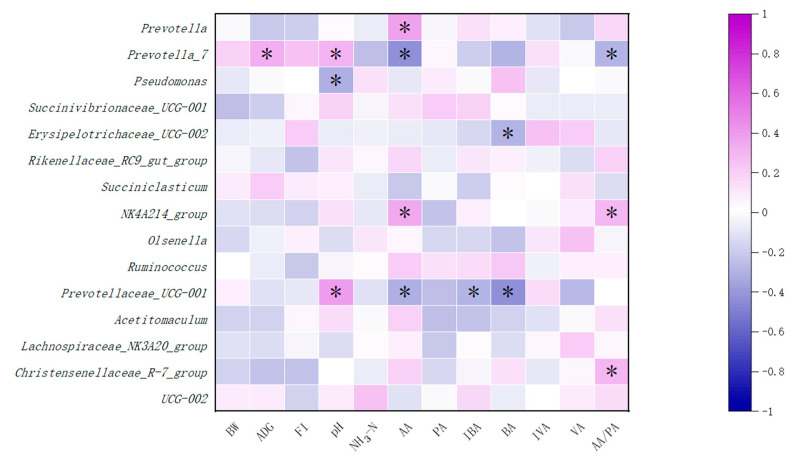
Correlation analysis between ruminal microorganisms and fermentation parameters. Numbers in the figure represent Pearson’s correlation coefficients, “*” stands for significant correlation at *p* < 0.05. BW, body weight; ADG, average daily gain at 1–84 d; FI, fecal index; NH_3_-N, ammonia nitrogen; AA, acetic acid; PA, propionic acid; BA, butyric acid; IVA, isobutyric acid; VA, valeric acid; IVA, isovaleric acid.

Pearson correlation analysis was carried out on the growth performance, fecal index, and ruminal fermentation parameters and the top 15 bacteria. As shown in Figure 4, *Prevotella-7* was positively correlated with ADG at 1–84 d in calves (*p* < 0.05). *Prevotella-7* and *Prevotella-UCG-001* were positively correlated with pH (*p* < 0.05), while *Pseudomonas* in *Proteobacteria* was negatively correlated with pH (*p* < 0.05). *Prevotella* and *NK4A214-group* were positively correlated with the concentration of acetic acid (*p* < 0.05), while *Prevotella-7* and *Prevotellaceae-UCG-001* were negatively correlated with the concentration of acetic acid (*p* < 0.05). *Erysipolitrichaceae-UCG-002* was negatively correlated with the concentration of butyric acid (*p* < 0.05). *Prevotellaceae-UCG-001* in *Bacteroidota* was negatively correlated with the concentration of butyric acid and isobutyric acid (*p* < 0.05).

## 4. Discussion

### 4.1. Dry Matter Intake and Growth Performance

Weaning is a critical period for calves, and growth in this period directly affects future production performance. Karami et al. [28] fed calves aged 70–120 days with diets containing different concentrate-to-forage ratios (50:50, 65:35, and 80:20), and found that decreasing the forage level increased the daily gain of calves linearly. Similarly, we also found that feeding 16.7% oat hay increased the ADG from calves fed oat hay as free-choice. The positive effect of forage on calf growth may be related to the improvement of the rumen environment and the enhancement of rumen muscle development [29], which in turn contributes to an increase in the solid feed intake in the late weaning period [30]. Meanwhile, feeding 16.7% oat hay increased the intake of protein and starch, which is also an important reason for the increased ADG of calves in our research. The feeding effect varies depending on the animal growth stage, environment, dosage of probiotics, strains, etc. Similar to the results of this experiment, the addition of CMP did not have significant effect on body weight and daily gain. Maamouri et al. [31] fed calves with 28 g/d of live yeast *Saccharomyces cerevisiae*, and found that the experimental group calves had a higher body weight and feed conversion rate, whereas Zhang et al. [32] supplemented with 1 × 10^8^ cfu/d *Lactobacillus plantarum* and *Bacillus subtilis* for calves, and no significant difference was observed regarding DMI and ADG. In our study, the results indicate that the addition of CMP has no significant effect on the body weight and daily gain of calves. The possible reason could be that the beneficial effects of probiotics would be observed only when the animals are not in good health.

### 4.2. Diarrhea Incidence

Pre-weaning and post-weaning are two critical stages for calves, in which they are particularly susceptible to intestinal infectious diseases [33]. The mortality rate of calves due to diarrhea and gastrointestinal disease is as high as 56.5%, causing significant economic losses for global dairy farms [34]. Karamzadeh-Dehaghani et al. [35] found that feeding calves with 3 g/d compound probiotics can effectively reduce the incidence of diarrhea and fecal score of calves. Stefańska et al. [36] fed calves with 250 mg/d multi-strain probiotics composed of *Lactobacillus casei*, *Lactobacillus salivarius*, and *Lactobacillus sake*, and found that the fecal score was lower than that of the control group, which is consistent with our results. We found that adding CMP can reduce the fecal index of calves, and the reason may be that probiotics can produce antibacterial compounds in vivo, such as hydrogen peroxide, organic acids, and bacteriocin [37]. These compounds can eliminate pathogenic bacteria, improve mucosal immunity, and enhance intestinal health by establishing beneficial intestinal flora.

### 4.3. Serum Antioxidant and Immune Indicators

The concentration levels of blood biochemicals can reflect the health and nutritional level of animals [38]. MDA is the product of lipid peroxidation of cell membranes, and its content indirectly reflects the production of free radicals and the degree of lipid peroxidation of cells [39]. Guo et al. [40] fed calves with different doses of multi-strain probiotics, and found that the content of MDA in calf serum was higher than that without probiotics, which is in agreement with our results. The MDA level in calf serum was decreased in the group with CMP, and the CAT activity was significantly higher than in the other groups. CAT is an antioxidant enzyme that removes hydrogen peroxide from the body and protects the mitochondrial membrane from destruction. The increase of CAT activity indicates that the body’s defense performance is stronger [41]. The IgA, IgG, and IgM contents were significantly higher in the group adding CMP than in the group without CMP, which was similar to the research of Wu et al. [42], suggesting that the CMP can improve the immunity of calves in this study. Moreover, feeding 16.7% oat hay increased the content of IgA, IgG, and IgM significantly in serum, which may be due to the increase in the intake of pelleted starter. The higher content of protein, vitamins, and trace elements in pelleted starter can improve the immune function of calves.

### 4.4. Rumen Fermentation Parameters

Rumen is the main digestive organ of ruminants and plays a key role in the normal growth of calves. Rumen pH, NH_3_-N, and VFA are critical indexes to assess rumen health. Llamas-Lamas et al. [43] found that when the proportion of alfalfa hay in the diet increased from 56% to 86%, the rumen pH value of dairy cows increased significantly. This is similar to the results of the present study, where there was a downward trend in the rumen pH values of calves in the 16.7% oat hay group compared to those in the oat hay free-choice feeding group. The reason may be that insufficient dietary fiber or lack of fiber effectiveness reduced the chewing time of ruminants, which would lead to decreased saliva secretion and decreased rumen pH [44]. Moreover, the higher intake of starch can easily ferment in the rumen to produce a large amount of VFA, leading to a decrease in rumen pH. Jiang et al. [45] fed calves with 1 × 10^10^ cfu *Lactobacillus plantarum* daily, and found that the concentration of butyric acid and microbial protein were increased. However, in our study, the effect of CMP on rumen fermentation parameters was not found, which may be due to the joint action of multiple strains or the difference of dairy calves. Different concentrate-to-forage ratios in ruminant diets affect the intake of energy and nonstructural carbohydrates, thus affecting rumen fermentation [46]. In this study, feeding 16.7% oat hay reduced the acetic acid concentration and the acetic acid to propionic acid ratio, and increased the propionic acid and valeric acid concentration. Consistent with the results of this study, Olijhoek et al. [47] fed dairy cows with dietary concentrate to forage ratios of 49:51, 70:30, and 91:9. The results showed that, with the increase of concentrate in the diets, the ruminal concentration of propionic acid of increased, while the concentration of acetic acid decreased. Therefore, when the ratio of concentrate to forage in the diet increased, the rumen fermentation pattern of dairy cows would change accordingly.

### 4.5. Rumen Bacteria Community

An increase of rumen microbial diversity promotes the stability of bacterial communities in the rumen ecosystem [48]. In this study, the ACE, Chao1, and Shannon indices of the CMP group increased significantly. The results showed that feeding CMP increased the richness and diversity of rumen bacteria in calves, which helped to maintain the balance and stability of the gastrointestinal bacteria. At the phylum level, the dominant microflora in rumen of weaned calves are *Firmicutes*, *Bacteroides,* and *Proteobacteria*, which is consistent with the previous research of Mao et al. [49]. *Firmicutes* participate in the degradation of cellulose, hemicellulose, starch, and oligosaccharides, which is closely related to energy conversion and harvest [50]. With an increasing proportion of forage in the diet, the relative abundance of *Firmicutes* also increased [51], which was consistent with the results of this experiment. The relative abundance of *Actinobacteria* is negatively correlated with a high-fat diet and positively correlated with fiber intake [52]. In this study, feeding 16.7% oat hay reduced the contents of *Actinobacterta* and *Actinobacteriota*, which may be related to the difference in dietary structure.

*Fibrobacterota* are the main bacteria for rumen fiber degradation, and can digest and ferment low-quality forage to produce VFA [53]. In this study, the addition of CMP showed a trend to increase the relative abundance of *Fibrobacterota*, suggesting that CMP could affect rumen fermentation. *Prevotella* has been proved to be involved in the production of VFAs, which are further used as energy by the host [54]. *Rikenellaceae-RC9-gut-group* may reduce the methane production by participating in VFA production and hydrogen scavenging [55]. *Ruminococcus* is ubiquitous in the human intestine and ruminant rumen microorganisms, and plays an important role in the fermentation of cellulose-rich feed and resistant starch [56,57]. Feeding CMP increased the relative abundance of *Prevotella*, *Rikenellaceae-RC9-gut-group*, *NK4A214-group,* and *Ruminococcus*. Fermentation of these known bacteria is helpful to stabilize rumen pH, reduce ammonia concentration, and improve fiber digestibility [58,59,60]. It has been reported that *Erysipolorichaceae-UCG-002* is related to VFA synthesis [61], but its specific function still needs to be explored. *Prevotella-7* often participates in the inflammatory reaction by producing redox proteins or increasing resistance to the host [62]. *Olsenella* is a beneficial bacterium among actinomycetes, and its abundance is limited by a high-fat diet [63]. In this experiment, feeding 16.7% oat hay reduced the abundance of *Prevotella*, *NK4A214-group,* and *Olsenella*, which may be related to higher intake of pelleted starter.

## 5. Conclusions

This study revealed that, compared with calves fed oat hay as free-choice, feeding a diet of 16.7% chopped oat hay increased the pelleted starter intake and ADG of dairy calves. Adding CMP to the pelleted starter resulted in lower diarrhea incidence, higher serum immunity and antioxidant capacity, bacterial diversity and richness, relative abundance of beneficial bacteria, and lower abundance of harmful bacteria in the rumen. Therefore, based on the results of this study, it is recommended to moderately limit the feeding of chopped oat hay and add CMP to the pelleted starter for calves aged 3–5 months.

## Figures and Tables

**Table 1:** Nutrient composition of pelleted starter and oat hay (% of DM) ^a^.

Nutrient Composition	Pelleted Starter ^b^	Oat Hay
Dry matter	90.56	89.45
NE_L_, MCal/kg	1.86	1.10
Crude protein	21.94	6.20
Starch	28.50	0.40
Ether extract	3.86	2.16
Neutral detergent fiber	21.82	63.93
Acid detergent fiber	8.15	32.71
Calcium	1.13	0.44
Phosphorus	0.52	0.15

^a^ Net energy of lactation (NE_L_) was a calculated value, and the others were measured values; ^b^ the pelleted starter consisted of 30.47% corn grain, 2.90% barley grain, 26.17% soybean meal, 2.50% double-low rapeseed meal, 17.48% wheat bran, 11.69% wheat middling, 2.25% cane molasses, 2.16% limestone, 0.22% dicalcium phosphate, 0.67% salt, 1.00% sodium bicarbonate, 0.22% magnesium oxide, 0.56% premix, and 1.72% L-lysine sulfate (dry matter basis). The premix was formulated to provide (per kilogram of dry matter) vitamin A 736 KIU, vitamin D_3_ 253 KIU, vitamin E 10,250 IU, vitamin K_3_ 0.23 g, vitamin B_1_ 0.23 g, vitamin B_2_ 0.575 g, vitamin B_6_ 0.345 g, vitamin B_12_ 2.76 mg, folic acid 0.115 g, biotin 13.8 mg, nicotinic acid 2.3 g, zinc 11,000 mg, manganese 8000 mg, iron 11,000 mg, copper 2250 mg, iodine 160 mg, cobalt 40 mg, and selenium 60 mg.

**Table 2:** Effect of dietary treatments on the growth performance of dairy calves.

Item ^a^	Treatment ^b^				SEM	*p*-Value ^c^		
	F	FP	L	LP		OH	CMP	OH × CMP
Pelleted starter intake (kg of DM/d)								
1–30 d	3.22	3.45	3.32	3.23	0.03	0.25	0.16	<0.01
31–60 d	4.24	4.43	4.61	4.52	0.03	<0.01	0.31	<0.01
61–84 d	4.99	4.72	5.13	4.98	0.03	<0.01	<0.01	0.22
1–84 d	4.15	4.20	4.38	4.24	0.03	0.04	0.51	0.17
Oat hay intake (kg of DM/d)								
1–30 d	0.56	0.65	0.58	0.58	0.01	0.29	0.05	0.05
31–60 d	1.01	1.02	0.85	0.89	0.02	<0.01	0.42	0.54
61–84 d	1.33	1.34	1.03	1.01	0.03	<0.01	0.74	0.42
1–84 d	0.97	1.01	0.82	0.85	0.02	<0.01	0.35	0.9
Total feed intake (kg of DM/d)								
1–30 d	3.78	4.10	3.90	3.81	0.03	0.17	0.05	<0.01
31–60 d	5.25	5.44	5.46	5.41	0.03	0.16	0.27	0.06
61–84 d	6.32	6.06	6.16	5.99	0.04	0.11	<0.01	0.51
1–84 d	5.12	5.20	5.20	5.09	0.04	0.86	0.91	0.28
Protein intake (g/d)								
1–30 d	741.02	796.14	763.67	744.96	6.31	0.21	0.11	<0.01
31–60 d	992.60	1034.53	1064.55	1046.70	6.58	<0.01	0.28	0.01
61–84 d	1177.65	1117.84	1189.39	1155.93	7.27	0.05	<0.01	0.30
1–84 d	970.53	983.10	1010.88	983.83	7.59	0.18	0.63	0.19
Starch intake (g/d)								
1–30 d	919.56	984.44	947.39	923.09	7.85	0.24	0.16	<0.01
31–60 d	1212.14	1266.15	1317.76	1291.26	8.57	<0.01	0.30	<0.01
61–84 d	1427.86	1349.32	1465.82	1424.42	9.60	<0.01	<0.01	0.23
1–84 d	1186.60	1200.08	1250.32	1213.01	9.44	0.04	0.52	0.17
NDF intake (g/d)								
1–30 d	1061.62	1167.26	1096.21	1077.60	10.95	0.16	0.03	<0.01
31–60 d	1570.18	1615.33	1550.03	1557.25	12.68	0.13	0.30	0.45
61–84 d	1940.23	1887.20	1780.53	1731.76	16.70	<0.01	0.04	0.93
1–84 d	1524.54	1558.58	1497.42	1468.88	15.99	0.04	0.71	0.48
Body weight (kg)								
0 d	93.67	93.58	93.5	94.08	0.78	0.92	0.88	0.84
30 d	132.67	135.58	133.67	135.17	1.14	0.90	0.35	0.76
60 d	162.5	168.0	167.42	166.42	1.29	0.53	0.39	0.21
84 d	189.33	191.92	193.75	195.42	1.36	0.15	0.44	0.87
ADG (kg/d)								
1–30 d	1.30	1.40	1.34	1.37	0.03	0.94	0.22	0.52
31–60 d	0.99	1.08	1.13	1.04	0.02	0.28	0.97	0.05
61–84 d	1.11	1.00	1.09	1.20	0.02	0.02	0.93	0.01
1–84 d	1.14	1.17	1.19	1.21	0.01	0.10	0.41	0.72

^a^ ADG, average daily gain. ^b^ F, oat hay fed as free-choice; FP, oat hay fed as free-choice, with 0.15% CMP added to pelleted starter; L, fed with a diet of 16.7% oat hay; LP, fed with a diet of 16.7% oat hay, with 0.15% CMP added to pelleted starter. ^c^ OH, oat hay; CMP, compound probiotics; OH × CMP, the interaction of OH and CMP.

**Table 3:** Effects of dietary treatments on the diarrhea incidence in dairy calves.

Item	Treatment ^a^				SEM	*p*-Value ^b^		
	F	FP	L	LP		OH	CMP	OH × CMP
Diarrhea rate, %	20.06	19.14	20.99	18.21	0.01	1.00	0.18	0.50
Fecal index	2.07	2.02	2.10	2.01	0.01	0.62	<0.01	0.23

^a^ F, oat hay fed as free-choice; FP, oat hay fed as free-choice, with 0.15% CMP added to pelleted starter; L, fed with a diet of 16.7% oat hay; LP, fed with a diet of 16.7% oat hay, with 0.15% CMP added to pelleted starter. ^b^ OH, oat hay; CMP, compound probiotics; OH × CMP, the interaction of OH and CMP.

**Table 4:** Effects of dietary treatments on the serum antioxidant and immune indexes in dairy calves.

Item ^a^	Treatment ^b^				SEM	*p*-Value ^c^		
	F	FP	L	LP		OH	CMP	OH × CMP
GSH-Px (nmol/L)	864.76	844.76	792.38	770.48	20.82	0.08	0.62	0.98
SOD (U/mL)	42.86	38.23	39.88	42.10	0.77	0.77	0.43	0.03
T-AOC (mmol/L)	0.86	0.80	0.88	0.86	0.02	0.31	0.22	0.54
CAT (U/mL)	1.95	2.24	1.85	2.69	0.08	0.21	<0.01	0.06
MDA (nmol/L)	5.76	3.84	4.60	4.80	0.18	0.74	<0.01	0.01
IgA (μg/mL)	1493.34	2029.98	1867.55	2395.00	67.21	<0.01	<0.01	0.96
IgG (mg/mL)	4.13	4.56	5.15	5.85	0.15	<0.01	0.02	0.56
IgM (μg/mL)	632.28	840.41	904.67	1063.90	37.15	<0.01	<0.01	0.69

^a^ GSH-Px, glutathione peroxidase; SOD, superoxide dismutase; T-AOC, total antioxidant capacity; CAT, catalase; MDA, malondialdehyde; IgA, immunoglobulin A; IgG, immunoglobulin G; IgM, immunoglobulin M. ^b^ F, oat hay fed as free-choice; FP, oat hay fed as free-choice, with 0.15% CMP added to pelleted starter; L, fed with a diet of 16.7% oat hay; LP, fed with a diet of 16.7% oat hay, with 0.15% CMP added to pelleted starter. ^c^ OH, oat hay; CMP, compound probiotics; OH × CMP, the interaction of OH and CMP.

**Table 5:** Effects of dietary treatments on the rumen fermentation parameters in dairy calves.

Item ^a^	Treatment ^b^				SEM	*p*-Value ^c^		
	F	FP	L	LP		OH	CMP	OH × CMP
pH	6.49	6.46	6.33	6.27	0.49	0.08	0.66	0.92
NH_3_-N (mg/dL)	10.94	11.62	11.95	12.10	0.58	0.54	0.73	0.83
TVFA (mmol/L)	84.00	88.38	87.02	85.85	1.91	0.94	0.60	0.37
Acetic acid (%)	67.38	65.88	62.50	62.95	0.72	0.01	0.70	0.48
propionic acid (%)	17.92	18.77	20.72	21.59	0.69	0.04	0.53	0.99
Butyric acid (%)	10.49	10.29	11.14	10.25	0.46	0.75	0.57	0.72
Isobutyric acid(%)	1.00	1.15	1.13	1.30	0.06	0.23	0.15	0.93
Valeric acid (%)	1.88	2.23	2.01	2.15	0.13	0.91	0.36	0.69
Isovaleric acid(%)	1.34	1.67	2.51	1.76	0.18	0.08	0.56	0.13
Acetic acid/propionic acid	3.96	3.74	3.15	2.89	0.18	<0.01	0.44	0.94

^a^ TVFA, total volatile acid. ^b^ F, oat hay fed as free-choice; FP, oat hay fed as free-choice, with 0.15% CMP added to pelleted starter; L, fed with a diet of 16.7% oat hay; LP, fed with a diet of 16.7% oat hay, with 0.15% CMP added to pelleted starter. ^c^ OH, oat hay; CMP, compound probiotics; OH × CMP, the interaction of OH and CMP.

**Table 6:** Effects of dietary treatments on the community diversity of rumen bacteria in dairy calves.

Item	Treatment ^a^				SEM	*p*-Value ^b^		
	F	FP	L	LP		OH	CMP	OH × CMP
Ace	1288.57	1380.35	1146.94	1224.26	21.99	<0.01	0.03	0.85
Chao1	1270.24	1367.42	1121.99	1206.28	22.57	<0.01	0.02	0.87
Simpson	0.96	0.94	0.90	0.95	0.01	0.08	0.40	0.03
Shannon	6.72	6.76	5.67	6.52	0.12	<0.01	0.03	0.05

^a^ F, oat hay fed as free-choice; FP, oat hay fed as free-choice, with 0.15% CMP added to pelleted starter; L, fed with a diet of 16.7% oat hay; LP, fed with a diet of 16.7% oat hay, with 0.15% CMP added to pelleted starter. ^b^ OH, oat hay; CMP, compound probiotics; OH × CMP, the interaction of OH and CMP.

**Table 7:** Effects of dietary treatments on the relative abundance of rumen bacteria flora in dairy calves (phylum level; %).

Item	Treatment ^a^				SEM	*p*-Value ^b^		
	F	FP	L	LP		OH	CMP	OH × CMP
*Firmicutes*	41.09	38.15	36.03	32.71	1.30	0.04	0.22	0.94
*Bacteroidota*	43.41	43.21	41.88	48.21	1.54	0.58	0.33	0.30
*Proteobacteria*	7.99	10.76	15.84	12.11	1.78	0.20	0.89	0.37
*Unidentified-Bacteria*	2.14	1.77	0.72	1.50	0.16	<0.01	0.49	0.06
*Actinobacteria*	0.25	0.78	0.19	0.05	0.10	0.05	0.31	0.10
*Actinobacteriota*	1.91	1.62	1.09	0.66	0.13	<0.01	0.12	0.77
*Euryarchaeota*	0.46	0.35	0.81	0.53	0.11	0.26	0.40	0.72
*Spirochaetota*	0.61	0.76	0.47	0.87	0.10	0.94	0.18	0.51
*Fibrobacterota*	0.08	0.14	0.07	0.31	0.04	0.37	0.08	0.32
*Desulfobacterota*	0.34	0.25	0.43	0.31	0.02	0.10	0.02	0.77

^a^ F, oat hay fed as free-choice; FP, oat hay fed as free-choice, with 0.15% CMP added to pelleted starter; L, fed with a diet of 16.7% oat hay; LP, fed with a diet of 16.7% oat hay, with 0.15% CMP added to pelleted starter. ^b^ OH, oat hay; CMP, compound probiotics; OH × CMP, the interaction of OH and CMP.

**Table 8:** Effects of dietary treatments on the relative abundance of rumen bacteria flora in dairy calves (genus level; %).

Item	Treatment ^a^				SEM	*p*-Value ^b^		
	F	FP	L	LP		OH	CMP	OH × CMP
*Prevotella*	15.81	18.06	7.97	15.91	1.35	0.05	0.05	0.27
*Prevotella-7*	13.87	4.49	22.58	10.16	1.78	0.02	<0.01	0.63
*Pseudomonas*	3.08	5.38	10.62	5.97	1.66	0.23	0.73	0.30
*Succinivibrionaceae-UCG-001*	4.23	4.80	4.97	5.92	1.00	0.65	0.71	0.93
*Erysipelotrichaceae-UCG-002*	6.61	4.92	6.29	1.97	0.63	0.18	0.02	0.28
*Rikenellaceae-RC9-gut-group*	3.79	4.89	2.78	6.66	0.46	0.66	<0.01	0.11
*Succiniclasticum*	4.89	3.11	4.41	4.15	0.35	0.69	0.15	0.28
*NK4A214-group*	1.48	2.63	0.63	1.45	0.18	<0.01	<0.01	0.60
*Olsenella*	1.79	1.46	1.06	0.63	0.13	<0.01	0.10	0.83
*Ruminococcus*	0.74	1.32	0.64	0.98	0.07	0.10	<0.01	0.36

^a^ F, oat hay fed as free-choice; FP, oat hay fed as free-choice, with 0.15% CMP added to pelleted starter; L, fed with a diet of 16.7% oat hay; LP, fed with a diet of 16.7% oat hay, with 0.15% CMP added to pelleted starter. ^b^ OH, oat hay; CMP, compound probiotics; OH × CMP, the interaction of OH and CMP.

## Data Availability

The original contributions presented in the study are included in the article. Further inquiries can be directed to the corresponding authors.
